# The impact of different polishing systems on yttria‐stabilized tetragonal zirconia polycrystalline crowns in relation to heat generation

**DOI:** 10.1002/cre2.778

**Published:** 2023-08-22

**Authors:** Xiaoyun Liu, John M. Aarts, Sunyoung Ma, Joanne Jung Eun Choi

**Affiliations:** ^1^ Sir John Walsh Research Institute, Faculty of Dentistry University of Otago Dunedin New Zealand

**Keywords:** heat generation, polishing, Y‐TZP, zirconia crown

## Abstract

**Objectives:**

To investigate the heat generation on yttria‐stabilized tetragonal zirconia polycrystalline (Y‐TZP) crowns during polishing with coarse and fine polishing systems at various speeds.

**Materials and Methods:**

Two polishers (coarse and fine) at three polishing speeds were investigated. Two simulation models of the first mandibular molars were prepared for full coverage Y‐TZP restorations with different reduction dimensions. Preheated water was pumped into the abutment chamber, to simulate the intrapulpal temperature and blood flow. Twelve Y‐TZP crowns (3M™ Lava™ Esthetic) were milled for each prepared tooth abutment and each cusp (*n* = 10) was individually ground for 30 s and polished for 2 min. Thermocouple wire was secured to the intaglio surface of the crown and linked to a data logger for recording temperature changes. Selected scanning electron microscopy (SEM) images of the treated surfaces and polishers were analyzed. The data was statistically analyzed using Prism 9.

**Results:**

The highest temperature rise was observed in the 20,000 RPM polishing speed groups for both coarse and fine polishing, and higher than the threshold value of 5.5°C for pulp damage. The Kruskal–Wallis test, revealed statistically significant differences (*p* < .0001) in heat generation between low (10,000 RPM) and high (20,000 RPM) polishing speeds.

**Conclusions:**

High‐speed polishing at 20,000 RPM generated the most heat over the threshold of 5.5°C, which would threaten the dental pulp. The results suggest that a cautionary approach should be taken to high‐speed intraoral polishing.

**Clinical Relevance:**

Dental clinicians should be aware of the choice of polishing systems and speeds to avoid pulp damage from intraoral polishing of Y‐TZP restorations.

## 1 INTRODUCTION

All‐ceramic crowns can deliver better esthetic results than metal alloy restorations, but they are not as durable as their metallic counterparts, particularly when used for posterior teeth. Generally, porcelain materials are brittle, and therefore they are prone to cracking. A scientific study first introduced and use of zirconia material in the medical field as a hip head prosthesis in 1969 (Helmer & Driskell, 1969). Yttria (Y_2_O_3_), as a dopant oxide, is commonly added for stabilizing the tetragonal phase of zirconia, which is known as the Yttrium‐stabilized tetragonal zirconia polycrystal (Y‐TZP) (Talibi et al., 2022). Y‐TZP replaced feldspathic ceramics is commonly used in the dental field as it does not rely on the luting cement for its strength (Nakamura et al., 2016). Y‐TZP is used in the dental field as all‐ceramic crowns due to its high tensile strength, hardness, and more recently due to high resistance to acid attack and corrosion (Madfa et al., 2014). Additionally, the introduction of CAD/CAM techniques promoted the development of Y‐TZP in dentistry because it simplified the manufacturing process (Boitelle et al., 2014).

Y‐TZP crowns are cemented using conventional cement or resin adhesives. If intraoral occlusal adjustment is done, it must then be polished. Intraoral polishing after adjustments is needed to remove plaque accumulation sites, improve patient comfort, decrease possibility of fracture caused by defects, and smooth occlusal surfaces in contact with opposing enamel to reduce wear of the natural tooth (Anusavice & Phillips, 2003; Chavali et al., 2017). A typical risk of friction during polishing procedures is heat generation, which is transferred to the tooth pulp chamber (Öztürk et al., 2004). A temperature rise of 5.5°C above body temperature (37°C) is the threshold temperature above which can have a detrimental effect on the pulp, resulting in inflammation and necrosis (Zach & Cohen, 1965). A heat increase (changes above 350°C) in the Y‐TZP material is also known to produce phase transformation, which has been shown to weaken Y‐TZP (Chavali et al., 2017). The heat generated from polishing Y‐TZP crowns may not always be high enough to induce phase transformation in the Y‐TZP, while even a moderate increase in temperature is sufficient to damage the pulp of a tooth (Chavali et al., 2017). Despite this concern the temperature increase from polishing Y‐TZP at different speeds has not been well reported previously.

It is accepted that in vivo studies are not an option since it is unethical and impossible to measure the temperature in the vital pulp cavity. However, most previous studies have been conducted in vitro, at room temperature and with ambient humidity, which is not replicating the intraoral temperature of 37°C. Despite this issue, in vitro studies carried out at room temperature did find a significant impact on temperature profiles (Chua et al., 2019; Dias et al., 2019; Kim, 2004). There is one previous study, which utilized an incubator at 37°C ± 1°C to simulate the pulp temperature, but it only simulated the intraoral temperature, not the temperature of the pulp chamber (Mathias et al., 2010). The pulpal blood flow is another influencing factor on the thermal behavior of the dentine‐pulp complex, which cannot be simulated by the stationary water inside a testing container (Chua et al., 2019). These issues raise a question about the validity of the methods and results from past studies and presents a significant opportunity for future research.

The aim of this study was to measure and evaluate the temperature difference induced in the pulp chamber during intraoral polishing for full‐contour Y‐TZP crowns using different polishing systems and speeds. The null hypotheses of this study were that there would be no difference in the heat production of Y‐TZP following different polishing systems and speeds.

## 2 MATERIALS AND METHODS

### 2.1 Specimen preparation

The mineralization, the thickness of dentine and enamel and even the thermal properties differ significantly among different teeth including gender, age, ethnicity, and between human deciduous and permanent teeth (Lancaster et al., 2017; Magalhães et al., 2008; Soori et al., 2022; Stroud et al., 1994; Wilson & Beynon, 1989). Therefore, this study utilized two plastic mandibular first molars as the abutment teeth for testing to eliminate experimental error. Before the tooth preparation, a polyvinylsiloxane matrix (3M™ Express™ Standard Putty Kit, 7312) was made, which was used to guide the amount to tooth reduction. One molar (Tooth 1) was reduced by 1.5 mm on the buccal cusps and 1.0 mm on the lingual cusps, while the other molar (Tooth 2) got an overall less than 1.0 mm reduction. The entire tooth reduction process was completed by utilizing diamond burs.

The scanning and restoration designs were accomplished by using an intraoral scanner (CEREC Primescan, Dentsply Sirona) and the “Biogeneric copy” design mode from CEREC SW 5.2.3. A total of 24 crowns (*n* = 12 per each tooth preparation) were then milled from full‐contour Y‐TZP discs (3M™ Lava™ Esthetic, 98S x 14 mm) by Ceramill Motion 2 with Ceramill Coolstream (Amann Girrbach). The milled Y‐TZP crowns were then sintered (Ceramill Therm) and glazed (IPS Ivocolor® Glaze Paste; Programat P310 G2 furnace, Ivoclar Vivadent). The crown material is Y‐TZP consisting of ZrO_2_ with 5 mol% Yttria and has an 800 MPa flexural strength.

### 2.2 Experiment set‐up

**Figure 1:**
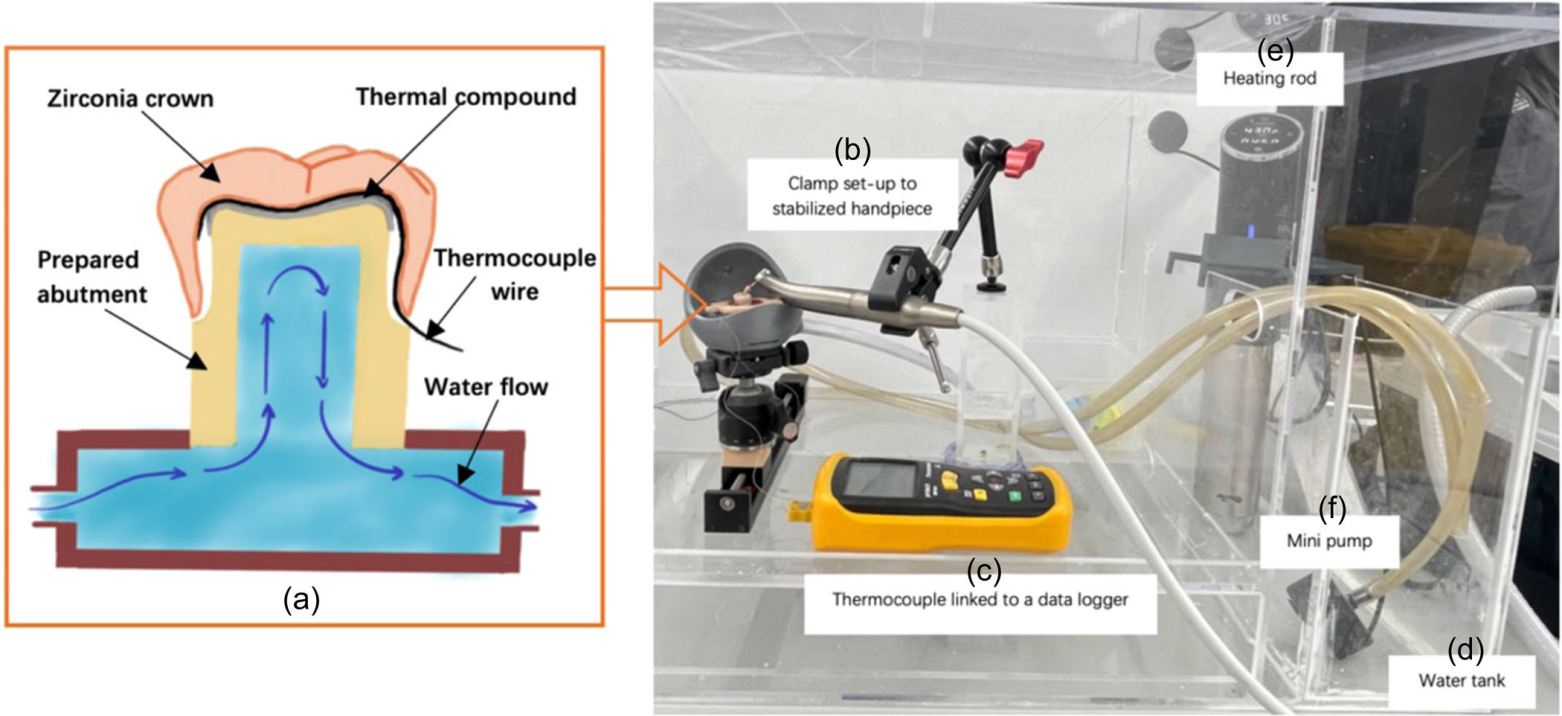
The simulating system of pulp temperature and blood flow, and the temperature testing apparatus. (a) Schematic diagram of the thermal compound applied on prepared abutment tooth, thermocouple wire underlying crown, and water flow inside the pulp chamber; (b) Clamp, with constant height, hold handpiece during grinding and polishing; (c) thermocouple data loggers to record the temperature changes; (d) water was heated up within a separate water tank; (e) Sous Vide Precision Cooker to heat the water; (f) submersible aquarium pump and tubes to transport water and simulate the pulp blood flow.

A previous study from Lau et al. (2023) investigated the changes in intrapulpal temperature during cavity preparation and utilized an oral simulation system. Then, the initial in vitro setup of the experiment was referred to this study. Before finalizing the test method one Y‐TZP crown was tested, this resulted a numerous modification to the testing chamber and testing model to achieve a workable model. The test model (Figure 1) had miniature K‐type thermocouple wires attached across the central groove of the preparation and connect with a thermometer (Protech QM1601, Electus Distribution). Furthermore, the thermocouple wire was secured on the occlusal aspect of the fitting surface of crowns in a S‐shape. This allowed for two segments of the wire to be across each cusp, which was equivalent to two detectors working together and compensates the probability of loose connection. The base of abutment molar was sealed in a printed jig by pattern resin (GC Australasia Dental, Australia) so it could be fixed over the water chamber. Water was warmed up in a tank by a heating rod (Kogan SmarterHome™ Sous Vide Precision Cooker) and pumped by a submersible aquarium pump into the chamber via a tube. A hole was drilled in the abutment tooth from the bottom towards the occlusal surface to allow for the warming water and ensure that sufficient material thickness of 1.0–1.5 mm was remaining (Figure 1).

To enhance the heatsink performance, a thin layer of nano diamond thermal compound (Duratech NM‐2018) was applied to the occlusal surface of the tooth preparation. The whole device was then sealed over the water chamber by heavy‐viscosity polyvinylsiloxane impression material (Flexitime Xtreme 2). One tube input the warm water to the chamber, the other tube discharged water back to the individual tank. Due to the heat loss during the water transportation, the heating rod in the tank was set at higher than 42°C until the thermometer reading showed 37°C. This circulatory system simulated the pulp temperature and blood flow. The last component within this set‐up was a holder with constant height used to clamp and stabilize handpieces (Figure 1).

A sample size calculation was performed by pilot study results using the software G*power v3.0.10 (Heinrich‐Heine‐Universita ¨t Düsseldorf). The effect size (*dz* = 4.9706) and the required sample size were calculated for *α* = .05 and a power of 0.95 (1‐β err prob), assuming a normal distribution. The calculation showed that 10 measurements per test group were required. For each abutment tooth, 12 mandibular first molar crowns were made each having five cusps, totaling 60 cusps, each cusp was utilized and counted as one sample unit. They were sequentially divided into six main groups, which were characterized by the polishers and polishing speeds. Each subgroup contained 10 crown cusps, shown in Table 1. Before polishing, occlusal surface was ground with a diamond bur at 30,000 RPM for 30 s to simulate an intraoral adjustment. This was done with a high‐speed handpiece (Ti‐Max Z95L, NSK) without water coolant. After a cooldown period, Meisinger coarse and fine (blue and orange) diamond polishers (Ivoclar Vivadent, 2‐step system for polishing zirconium oxide restorations), were used at a rotatory speed of 10,000, 15,000, and 20,000 RPM for 2 min without coolant. This was mounted to a handpiece (T2 Line A 40L, Dentsply Sirona) and a micromotor (NSK VIVA ace). Even though the recommended maximum polishing time from the polisher manufacturer (Ivoclar Vivadent) is 60‐s, 2‐min time period was conducted for the further analysis in the correlation between the continuous polishing and heat accumulation. The polisher was replaced every five cusps (one crown) and the testing was arranged in an order from the mesiobuccal cusp, distobuccal cusp, distal cusp, distolingual cusp, to mesiolingual cusp.

**Table 1:** List of test groups by polishing system and polishing speed (total *n* = 120).

Groups	Polisher used	Rotation speed in RPM	Tooth 1 numebr of specimens	Tooth 2 number of specimens
C10	Meisinger coarse polisher (DCA04 204 040)	10,000	10	10
C15	Meisinger coarse polisher (DCA04 204 040)	15,000	10	10
C20	Meisinger coarse polisher (DCA04 204 040)	20,000	10	10
F10	Meisinger fine polisher (DCA10 204 040)	10,000	10	10
F15	Meisinger fine polisher (DCA10 204 040)	15,000	10	10
F20	Meisinger fine polisher (DCA10 204 040)	20,000	10	10

Abbreviations: C, coarse polishing; F, fine polishing; 10: 10,000 RPM; 15: 15,000 RPM; 20: 20,000 RPM.

### 2.3 Measurement of heat generation

Each time a crown was replaced for a new test, the temperature setting of the heating rod was calibrated until the reading on the thermometer connected to thermocouple was as close to 37°C without fluctuation. The EasyLog USB thermocouple data logger was used and data was downloaded in graphical format and exported to Excel. The mean, maximum, and minimal temperature values for each testing group were then calculated. The heat generation was also determined by the formula as “(maximum temperature – initial temperature).” The initial temperature was recorded because it was not the same for each group, therefore recording this enabled a more accurate temperature change measurement to be recorded and allowed for further statistical analysis.

### 2.4 Statistical analysis

Before any statistical comparisons were made, correlation tests were performed on the data sets using a Shapiro–Wilk test and a Kolmogorov‐Smirnov test by PRISM 9 to assess the normality. The temperature data sets were statistically analyzed using a Kruskal–Wallis test. A *p*‐value of <0.05 was considered statistically significant.

Comparisons were made on the change of temperature profiles between (1) coarse and fine polishing with the same speed, (2) different polishing speeds with the same polisher and crown, and (3) crowns from two different prepared abutments with the same polishing and speed.

### 2.5 SEM analysis

The treated crowns including the new and worn polishers, were mounted on aluminum stubs using double‐sided carbon tape and carbon paste, and coated with 15 nm of gold palladium (Quorum Q150V Plus modular coating system, Quorum Technologies Limited). The SEM images were captured using a Zeiss Sigma 300VP FESEM (Carl Zeiss Inc) at an accelerating voltage of 5 KV.

## 3 RESULTS

### 3.1 Heat generation

**Figure 2:**
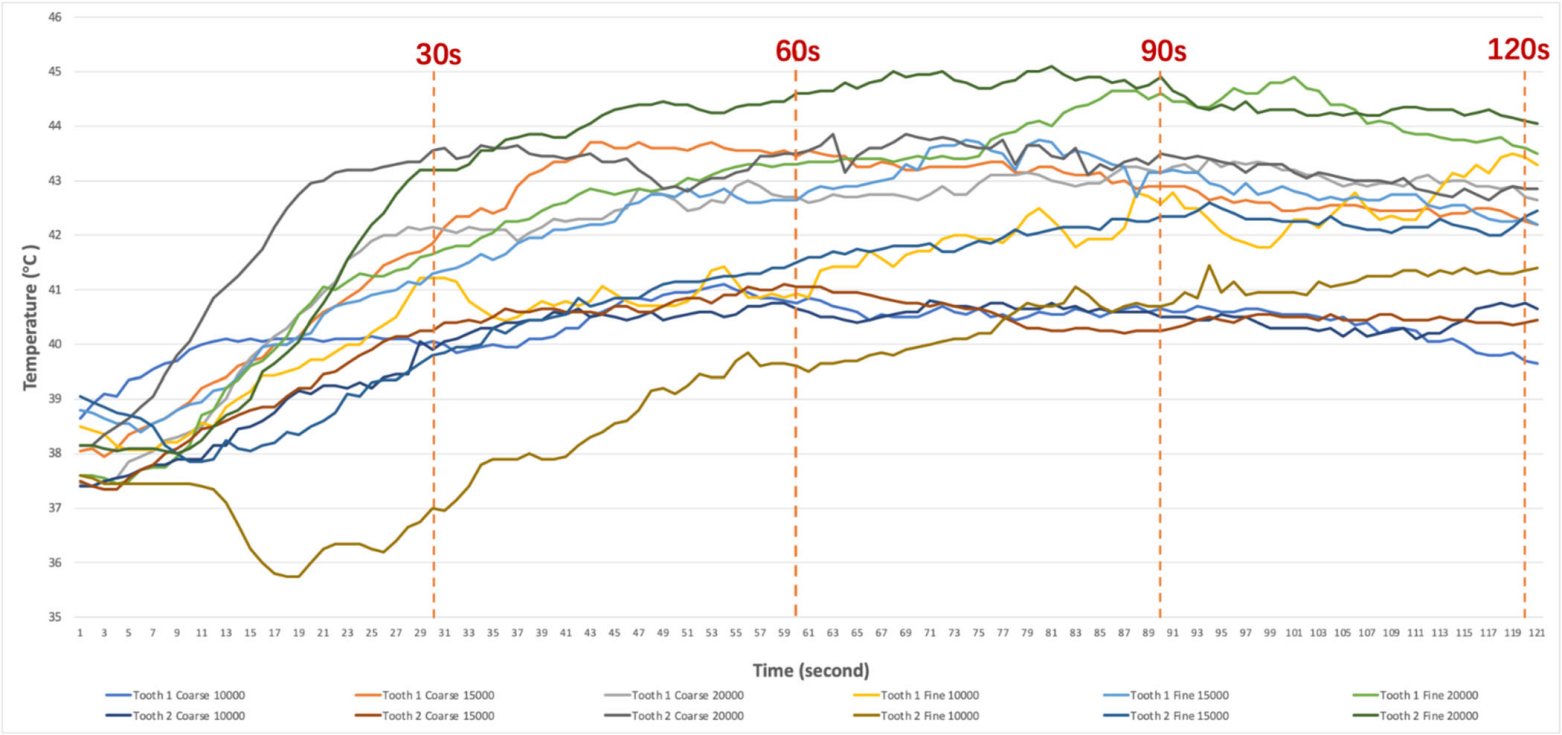
Overall mean temperature change for each group in a polishing period of 2 min. Dotted line indicates the every 30‐s.

The average values of initial, maximum, and minimum temperatures (±SD) for different polishing groups are summarized in Table 2. Figure 2 shows the mean temperature changes over time. When looking at the results in Figure 2, the 60‐s point is indicated, which is the recommended polishing time from the manufacturer (Ivoclar Vivadent). For all polishing groups, the temperature dropped within the first 20‐s period. Table 3 shows the average values of maximum temperatures (±SD) and heat generation within first 60 s for each group. Compared to the values in Table 2, all groups resulted in a lower temperature rise values within the recommended polishing time (60 s) (Table 3). As the temperature rise threshold is 5.5°C, the maximum heat generation of most polishing groups at 10,000 and 15,000 RPM (except for C15, F10, F15 of Tooth 1) did not exceed the safe threshold for a tooth pulp. However, the fine polishing at 10,000 and 15,000 RPM for the crown on Tooth 1 did not see the temperature rising above the safe threshold during the first 60 s. Both crown designs under coarse and fine polishing at 20,000 RPM resulted in temperature rises of at least 6°C within first 60 s and 8°C within 2 min. For the crown on Tooth 1 (with 1.0–1.5 mm tooth reduction), the amount of heat generation increased as the polishing speed increased. However, the result for the crown on Tooth 2 (with <1.0 mm tooth reduction) at 15,000 RPM polishing speed caused the lowest temperature rise.

**Table 2:** The mean (±SD) of initial, maximum and minimum temperatures, and mean heat generation within 2 min for different groups.

Tooth 1						
Specimen group	C10	C15	C20	F10	F15	F20
Initial temp (°C)	38.65 ± 0.82	38.05 ± 0.93	37.45 ± 1.21	38.50 ± 0.50	38.80 ± 0.67	37.60 ± 0.77
Maximum temp (°C)	42.15 ± 2.77	44.95 ± 5.60	45.85 ± 4.58	44.21 ± 4.62	45.15 ± 4.16	46.05 ± 4.27
Minimum temp (°C)	38.00 ± 0.82	37.60 ± 0.93	37.20 ± 1.21	37.86 ± 0.50	38.15 ± 0.67	37.30 ± 0.77
Heat generation (°C)	3.50	6.90	8.40	5.71	6.35	8.45
Tooth 2						
Specimen group	C10	C15	C20	F10	F15	F20
Initial temp (°C)	37.40 ± 0.39	37.50 ± 0.58	38.15 ± 1.40	37.60 ± 0.70	39.05 ± 0.55	38.15 ± 0.24
Maximum temp (°C)	42.50 ± 1.86	42.00 ± 1.83	46.15 ± 5.96	42.95 ± 1.82	43.45 ± 1.92	46.40 ± 2.66
Minimum temp (°C)	37.15 ± 0.39	37.20 ± 0.58	38.05 ± 1.40	35.45 ± 0.70	37.35 ± 0.55	37.70 ± 0.24
Heat generation (°C)	5.10	4.50	8.00	5.35	4.40	8.25

Abbreviations: C, coarse polishing; F, fine polishing; SD, standard deviation; temp, temperature; 10: 10000 RPM; 15: 15000 RPM; 20: 20000 RPM.

**Table 3:** The mean (±SD) of initial and maximum temperatures, and mean heat generation within first 60 s for different groups.

Tooth 1						
Specimen group	C10	C15	C20	F10	F15	F20
Initial temp (°C)	38.65 ± 0.82	38.05 ± 0.93	37.45 ± 1.21	38.50 ± 0.50	38.80 ± 0.67	37.60 ± 0.77
Maximum temp (°C)	41.85 ± 2.95	44.35 ± 5.82	44.45 ± 4.82	42.07 ± 3.52	43.85 ± 3.26	43.95 ± 3.26
Heat generation (°C)	3.20	6.30	7.00	3.57	5.05	6.35
Tooth 2						
Specimen group	C10	C15	C20	F10	F15	F20
Initial temp (°C)	37.40 ± 0.39	37.50 ± 0.58	38.15 ± 1.40	37.60 ± 0.70	39.05 ± 0.55	38.15 ± 0.24
Maximum temp (°C)	41.55 ± 2.01	41.65 ± 1.97	44.85 ± 6.81	40.50 ± 1.63	41.90 ± 2.09	45.15 ± 3.82
Heat generation (°C)	4.15	4.15	6.70	2.90	2.85	7.00

Abbreviations: C, coarse polishing; F, fine polishing; SD, standard deviation; temp, temperature; 10: 10000 RPM; 15: 15000 RPM; 20: 20000 RPM.

**Figure 3:**
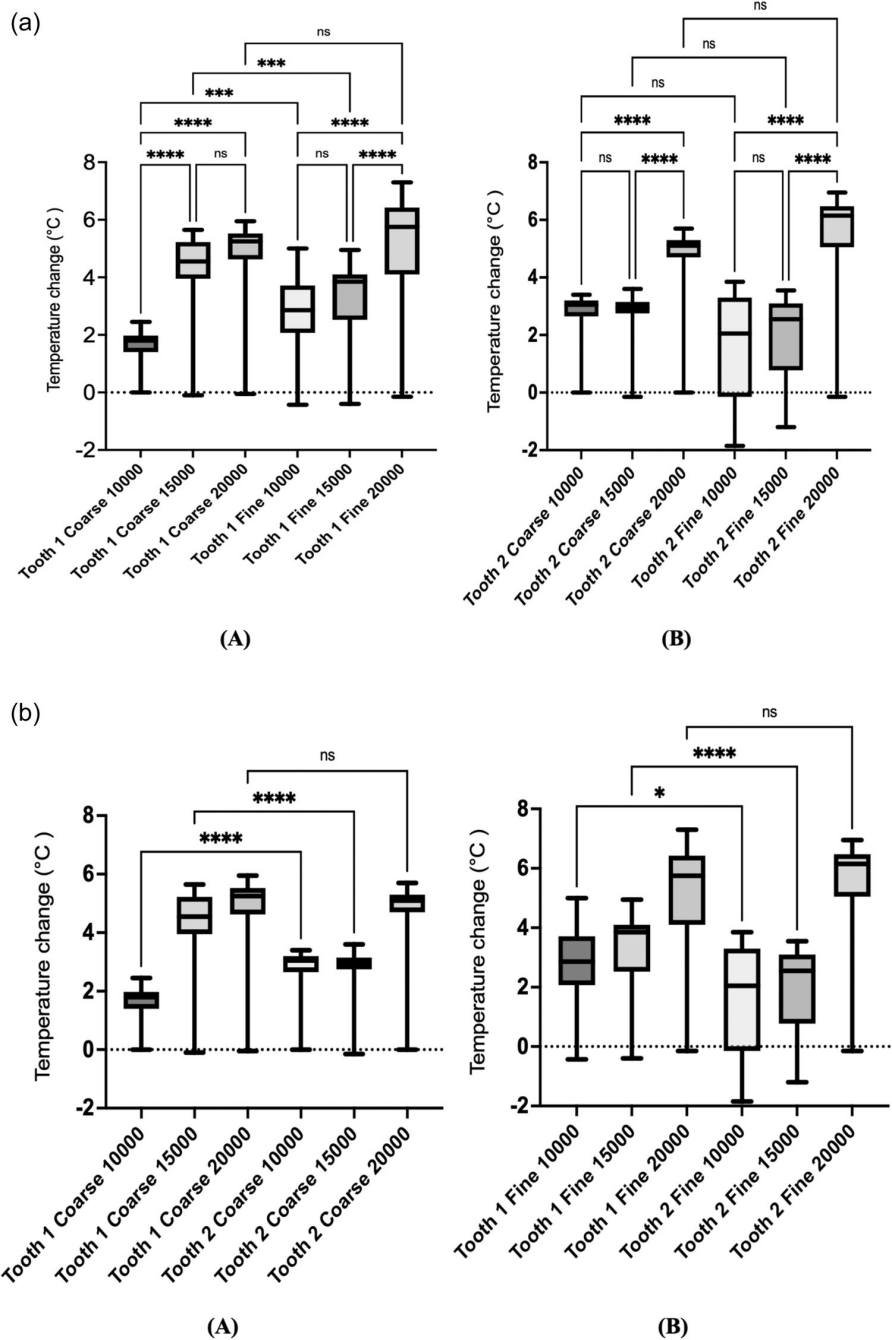
(a) Comparison of polishers or polishing speeds within the same crown. Asterisks represent *p*‐value classification; “ns” represent no significant. (b) Comparison between two crowns with different prepared reduction. Asterisks represent *p*‐value classification; “ns” represent no significant.

Within the two respective crown design groups (Tooth 1 and 2), the multiple comparisons between coarse and fine polishings, as well as among the various polishing speeds are shown in Figure 3a. Statistically significant differences were observed between the 10,000 RPM and 20,000 RPM speeds for both coarse and fine polishers, as well as between the two different crown thicknesses (*p* < .0001). Except for coarse polishing of crown 1 (*p* = .2376), there was a statistical difference between 15,000 RPM and 20,000 RPM speeds (*p* < .0001). The coarse polishing of the crown on Tooth 1 did show a significant difference (*p* < .0001) when compared to 10,000 RPM and 15,000 RPM speeds. No statistically significant difference was observed between coarse and fine polishing of the crown on Tooth 2 (*p* > .05), while these two polishers showed a statistical difference at 10,000 RPM or 15000 RPM within the results for the crowns on Tooth 1 (*p* < .0001).

The comparisons between the two crowns for the two different tooth reductions is shown in Figure 3b. No significant difference was observed between crown 1 and 2 for either coarse or fine polishing at 20,000 RPM speed (*p* > .99). However, statistical differences were found within both 10,000 and 15,000 RPM speeds (*p* < .0001), except for in Tooth 1 F10 compared to Tooth 2 F10 (*p* = .0138).

### 3.2 SEM analysis

**Figure 4:**
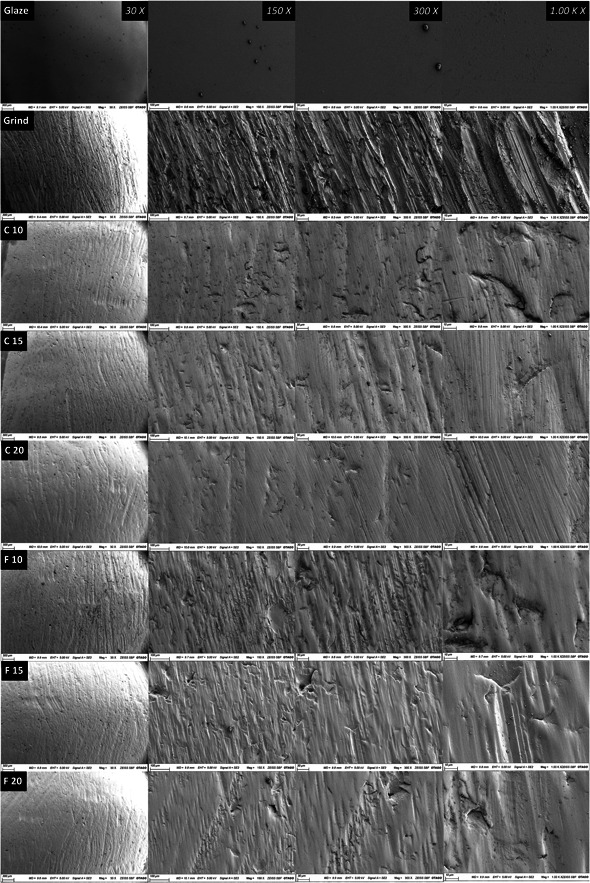
Scanning electron microscopy (SEM) images of the zirconia crown surfaces after different treatments (glazing, grinding, and coarse and fine polishings). C, coarse polishing; F, fine polishing; 10: 10,000 RPM; 15: 15,000 RPM; 20: 20,000 RPM.

The surface textures of Y‐TZP crowns after various surface treatments are shown in Figure 4. Glazing resulted in a mirror‐like surface topography with little glaze paste granules remaining. After grinding, the scuff and marks from the abrasive diamond bur were obvious, even under low magnification (Figure 4, Grinding‐30X). Most deep grooves on the crown surfaces were present in the same direction of the grinding (Figure 4, Grinding‐150X). Both coarse and fine polishing smoothened and flattened the rough surface. Higher polishing speed removed more scratches. After coarse polishing, grooves resulting from griding appeared to be shallower and appeared blunted, as seen in groups C10, C15, and C20 at all magnifications (Figure 4), whereas they were more frequent in quantity in all crowns that were polished with fine polishers at 10,000, 15,000, and 20,000 RPM polishing speeds, at all magnifications (Figure 4). Coarse polishers showed effective removal of deep pits and fissures created by grinding as seen in Figure 4 image C20 ‐1.00K X (the higher the speed, the more effective removal of high points), whereas the fine polishers showed less effective removal of the ground pits even at higher speed, as seen in Figure 4 image F10, 1.00K X.

**Figure 5:**
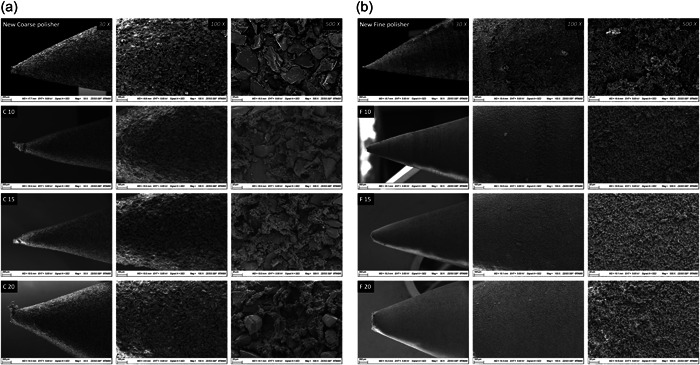
(a) Scanning electron microscopy (SEM) images of the new and used coarse polishers. C, coarse polishing; 10: 10,000 RPM; 15: 15,000 RPM; 20: 20,000 RPM. (b) SEM images of the new and used fine polishers. F, fine polishing; 10: 10,000 RPM; 15: 15,000 RPM; 20: 20,000 RPM.

Figure 5a,b show the comparison between new and used polishers. The abrasive particles were roughly 20–50 μm in the Meisinger coarse polisher and 5–10 μm in the Meisinger fine polisher. As the polishing speed increased, the wear and distortion of polisher tips became more apparent. The diamond particles of the polisher were worn, especially around the tip areas (working part), and some of them were pulled out from the matrix (Figure 5a,b, C10, C15, C20, F10, F15, F20‐500X).

## 4 DISCUSSION

This in vitro study aimed to assess the temperature changes during coarse and fine polishings of Y‐TZP crowns. Two common types of abutment preparation for Y‐TZP restorations were investigated. One was with buccal cusp reduction of 1.5 mm and lingual cusp reduction of 1.0 mm, and the other was reduced with overall less than 1.0 mm. The results exhibited a significant difference in heat generation between most of the various polishing speeds and two different tooth reductions at 10,000 and 15,000 RPM speeds. Therefore, the null hypotheses that there would be no statistically significant difference in heat production by coarse and fine polishers and at three polishing speeds were rejected.

There are a few potential limitations within this study that need to be considered. First, as the current study is in vitro and not human teeth, the real consequence in the clinical practice might be slightly different. This could be influenced by cement material, remaining enamel thickness, and relative humidity. Additionally, various polishing systems and different applied forces may influence heat generation. Therefore, further study could focus on these topics and assess the correlation between them and temperature changes.

The fundamental mechanism of dental polishing is utilizing abrasive particles to properly remove the material or smooth the surface (Jefferies, 2007; Remond et al., 2002). As the crown surface and rotating polisher is subjected to a relative tangential motion, the frictional energy within the contact interface is dissipated and transformed to frictional heat (Amiri & Khonsari, 2010; Kisuka et al., 2021). This consequently presents as the local temperature rise. It could be influenced by both experimental factors, such as speed, duration and applied force and environmental conditions. Across all groups, polishing at 20,000 RPM increased the temperature the most, increasing the temperature by more than 8°C which was higher than the safe threshold level (5.5°C) (Zach & Cohen, 1965). This indicates it could cause pain, pulp damage or necrosis for patients, even though the speed is the maximum speed recommended by the manufacturers. Slowing the polishing speed by 10,000 RPM, resulted in the heat generation being significantly reduced. This can be easily explained as the frequency of sliding movements of the polisher over the surface of the crown is effectively halved during the same duration.

Except for the rotational velocity of polishers, the polishing duration represents another critical factor for accumulating heat on the restoration surface. As shown in Tables 2 and 3, the maximum heat generation was unreached within the recommended polishing time from the manufacturer (1 min). It indicates that heat would be continuously built up and transmitted through Y‐TZP crown, which is time‐varying. However, it is apparent that the temperature increase is not infinite and the maximum temperature in general leveled out after the first 60 s and then fluctuated around that level. This can be explained as the heat conduction between different objects happens when there is a temperature difference (Bergman et al., 2011). As the motion of the polisher can only produce a certain level of heat and Y‐ZTP has a low thermal conductivity, the temperature gradient remained relatively steady. Only the fine polishing at 10,000 RPM resulted in a constant elevation in temperature, this could reflect the slower rate of temperature climb at low‐speeds, however, at the 2‐min point the temperature was similar to the other groups.

A study by Chavali et al. (2017) of four polishers from two brands (CeraMaster and Dialite ZR) and three polishing speeds (5000, 15,000, and 40,000 RPM) reported that the highest speed, coarse, medium or fine polishing, for 1 min, elevated the temperature by over 10°C. This was higher than what was found in the current study. This could reinforce that the temperature levels produced by various polishers will increase with the corresponding increase in velocity of polisher rotation. Chavali et al (Chavali et al., 2017) also assessed a specimen thickness of 1 mm which was similar to Tooth 2, while coarse and fine polishings resulted in higher heat generation compared to this study. The potential explanation might be that Chavali et al. (2017) did not use a water circulation system, and therefore their results might not have accounted for the intra‐pulp temperature and the blood flow that could have allowed better control of the temperature of the pulp chamber.

In terms of heat generation in different Y‐TZP crown thickness, there was no statistical difference (*p* > .9999) between the crowns made on Tooth 1 and 2 during coarse and fine polishings at 20,000 RPM. For coarse polishing at 10,000 RPM, a relatively higher temperature was observed and could have been a consequence of a thinner crown thermal transmission allowing heat transfer easier and faster across a shorter distance. The results of rotational velocity of 15,000 RPM were inconsistent, as the temperature rise is inversely proportional to the occlusal restoration thickness. One possible reason is the slight difference in environmental conditions, since not all tests proceeded on the same day. With the changes in weather, the ambient humidity varies. It is evident that an increase in relative humidity leads to a slight decrease in the coefficient of friction (Lian et al., 2018). This is an important parameter that influences heat generation from a friction process (Kisuka et al., 2021). Another potential cause may be from the heat that would escape rapidly through a thin‐layer material rather than a thicker layer (Buchanan, 2020). The frictional heat during surface finishing penetrates into and accumulates within the crown, and the heat is then transferred to the outer surface by thermal conduction and finally lost to the surroundings by thermal radiation and convection (Carpenter & Kissock, 2006).

This study found that different polishing protocols resulted in a temperature increases in Y‐TZP crowns, however, the end temperature increase within a natural tooth may be less. This is because the thermal conductivities of the remaining enamel and dentine after tooth reduction, as well as the cementation material, are lower than Y‐TZP (Anusavice & Phillips, 2003; Lancaster et al., 2017). Therefore, they would perform as a barrier to impede heat transferred to the pulp chamber and prevent pulp damage. Differences in enamel thickness have been found between the sexes, among populations, and even between the upper and lower jaw arches (Smith et al., 2006). Thus, its protective effect can vary among individuals.

According to the SEM analysis, chipping of diamond abrasives on the polishing burs were evident and generated lots of deep grooves on the crown surface. The rugged surface would result in varying light reflection and lack of luster and shine. This detrimental effect would be compensated by the progressive polishing process, as it could slightly flatten the rough surface. In clinical practice, heat generation is a crucial parameter to maintain a healthy pulp. Even though the highest speed at 20,000 RPM in the current study resulted in a smoother surface, it may cause pulpal inflammation and necrosis. The positive correlation of polishing speeds and duration with heat generation during the process might be always the case for other dental materials, such as gold alloy or glass ceramic. The different thickness of remained dentine layer would also influence how much protection there is to the pulp from the heat generated. Even though the present study utilized a typodont molar model and focused on zirconia, the results indicated that the clinicians should pay close attention to the heat produced by intraoral polishing and avoid damaging the dental pulp. To avoid potentially excessive heat generation, it is recommended to polish at a low speed over an extended period of time, or alternatively polish at a higher speed, but allow time interval for cooling. Future study could involve other dental materials and investigate the influence of remained tooth structure which can provide more comprehensive guidance to the dental practitioners.

## 5 CONCLUSIONS

Within the limitations of this in vitro study, the following conclusions were made:

1. Coarse and fine polishing at 20,000 RPM generated the most heat that went over the threshold of 5.5°C and could potentially damage the dental pulp.
2. A polishing speed of 10,000 RPM showed significantly lower (*p* < .0001) heat generation than 20,000 RPM polishing speed.
3. Heat transferred through thicker and thinner Y‐TZP crowns was only significantly different during polishing at 10,000 and 15,000 RPM.

## AUTHOR CONTRIBUTION

**Xiaoyun Liu**: Conceptualization; literature search; investigation; data collection, interpretation; statistical analysis; writing and reviewing of manuscript. **John Aarts**: Supervision; critical revisions; writing and reviewing of manuscript. **Sunyoung Ma**: Supervision; reviewing of manuscript draft. **Joanne Jung Eun Choi**: Oversight and coordination of overall project; conceptualization; data interpretation; supervision; writing of manuscript.

## CONFLICT OF INTEREST STATEMENT

The authors declare no conflict of interest.

## Data Availability

Data will be available upon request.
